# Epidemiology and Antifungal Susceptibility of *Candida* Species Isolated from 10 Tertiary Care Hospitals in Iran

**DOI:** 10.1128/spectrum.02453-22

**Published:** 2022-11-29

**Authors:** Parisa Badiee, Teun Boekhout, Pardis Haddadi, Rasoul Mohammadi, Abdolkarim Ghadimi-Moghadam, Jafar Soltani, Ali Zarei Mahmoudabadi, Seyyed Amin Ayatollahi Mousavi, Mohammad Javad Najafzadeh, Kambiz Diba, Ali Reza Salimi-Khorashad, Maneli Amin Shahidi, Fatemeh Ghasemi, Hadis Jafarian

**Affiliations:** a Clinical Microbiology Research Center, Shiraz University of Medical Sciences, Shiraz, Iran; b Westerdijk Fungal Biodiversity Institute, Utrecht, The Netherlands; c Institute for Biodiversity and Ecosystem Dynamics, IBED, University of Amsterdam, Amsterdam, The Netherlands; d Department of Periodontology, Faculty of Dentistry, Lorestan University of Medical Sciences, Khorramabad, Iran; e Department of Medical Parasitology and Mycology, School of Medicine, Infectious Diseases and Tropical Medicine Research Center, Isfahan University of Medical Sciences, Isfahan, Iran; f Department of Pediatrics Infectious Disease, Emmam Sajjad Hospital, Yasuj University of Medical Sciences, Yasuj, Iran; g Department of Pediatrics, Faculty of Medicine, Kurdistan University of Medical Sciences, Sanandaj, Iran; h Infectious and Tropical Diseases Research Center, Health Research Institute, Ahvaz Jundishapur University of Medical Sciences, Ahvaz, Iran; i Department of Medical Parasitology and Mycology, Faculty of Medicine, Kerman University of Medical Sciences, Kerman, Iran; j Department of Medical Parasitology and Mycology, Mashhad University of Medical Sciences, Mashhad, Iran; k Cellular and Molecular Research Center, Urmia University of Medical Sciences, Urmia, Iran; l Department of Parasitology and Mycology, School of Medicine, Infectious Diseases and Tropical Medicine Research Center, Zahedan University of Medical Sciences, Zahedan, Iran; University at Albany, State University of New York

**Keywords:** *Candida* species, caspofungin, voriconazole, amphotericin B, isavuconazole, itraconazole, luliconazole, posaconazole, fluconazole, Iran

## Abstract

In recent decades, the incidence of *Candida* infections has increased in immunocompromised patients. This multicenter study aimed to evaluate *in vitro* antifungal activities of 8 antifungal agents against the *Candida* species isolated from 10 university hospitals in Iran. During the period from Dec 2019 to Dec 2021, *Candida* species were collected from clinical samples of patients. The isolates were identified by PCR restriction fragment length polymorphism and sequencing methods. The antifungal susceptibility tests of each isolate to eight antifungal agents were performed according to the microdilution CLSI M27, M59, and M60 standard methods. A total of 598 *Candida* strains were isolated from clinical samples. The most commonly isolated *Candida* species was C. albicans, followed by C. glabrata, C. parapsilosis, Debaryomyces hansenii (Candida famata), C. tropicalis, *Pichia kudriavzevii* (Candida krusei), C. orthopsilosis, Meyerozyma guilliermondii (Candida guilliermondii), Kluyveromyces marxianus (Candida kefyr), and Clavispora lusitaniae (Candida lusitaniae). MIC_90_ values in all *Candida* species were as follows: 0.25 μg/mL for caspofungin and voriconazole; 0.5 μg/mL for amphotericin B and isavuconazole; 2 μg/mL for itraconazole, luliconazole, and posaconazole; and 16 μg/mL for fluconazole. Although 30/285 C. albicans, 15/31 *C. hansenii,* 3/12 *M. guilliermondii*, 67/125 C. glabrata, 5/15 *P. kudriavzevii*, 6/60 C. parapsilosis, and 5/23 C. tropicalis isolates were multiazole resistant with resistance to 2 to 4 azoles, pan-azole resistance was not observed. According to our data, Candida albicans and C. glabrata were the most frequent species isolated from clinical samples in Iran. Caspofungin and voriconazole, with lower MIC_90_ values, are the most effective than other antifungal agents for the treatment of *Candida* infections in this region.

**IMPORTANCE** *Candida* species cause severe invasive infections of the heart, brain, eyes, bones, and other parts of the body. Knowledge of regional distributions of causative *Candida* agents and their antifungal susceptibility patterns can help to monitor resistance to antifungal agents of various species and support local and national surveillance programs. In the present study, C. albicans and C. glabrata were the most frequently isolated species from clinical samples in Iran. Increasing rates of non-*albicans Candida* isolates from the Iranian population should be looked at as alarming due to various levels of intrinsic MIC values or resistance to various antifungal drugs. Caspofungin and voriconazole are recommended over fluconazole for the treatment of *Candida* infections in the study region. However, amphotericin B and isavuconazole are also active against the most common *Candida* species isolated from patients. Pan azole-resistant *Candida* species were not observed in the present study.

## INTRODUCTION

*Candida* species are commensal yeasts occurring on human mucous membranes (vagina and oral cavities), in the gastrointestinal tract, and on the skin (1). The species cause severe invasive infections of the heart, brain, eyes, bones, and other parts of the body, especially cutaneous and mucosal parts (1, 2). In recent decades, the incidence of *Candida* infections has increased due to the growing number of patients suffering from leukemia, bone marrow and solid organ transplantation, diabetes mellitus, HIV, and those receiving immunosuppressive drugs (1–4). Candidemia is the third or fourth most common causative agent of bloodstream infection in hospitalized patients (5). Early diagnosis and effective therapy result in the best management of the respective patients. According to the literature, antifungal therapy applied within 24 h of candidemia onset decreases the mortality rate to 52.8% (*n* = 142), compared to 97.6% (*n* = 82) in patients not receiving antifungal therapy (6). Candida albicans is the most frequent cause of *Candida* infections, although other species such as C. glabrata, C. parapsilosis, and C. tropicalis have been reported (7, 8). Prolonged treatment may induce mutations conferring resistance of *Candida* species to the various antifungal agents (3, 9). Resistance to echinocandins is low, but prolonged use of it results in elevated mean inhibition concentrations (MICs) of echinocandins for several species (9–11). Determining the epidemiology of clinically relevant *Candida* species and their susceptibility patterns is important for monitoring the treatment efficacy and the emergence of resistance. The aim of this multicenter study was to evaluate *in vitro* antifungal activities of 8 antifungal agents (i.e., azoles, echinocandins, and amphotericin B) in *Candida* species isolated from 10 university hospitals in Iran.

## RESULTS

**FIG 1:**
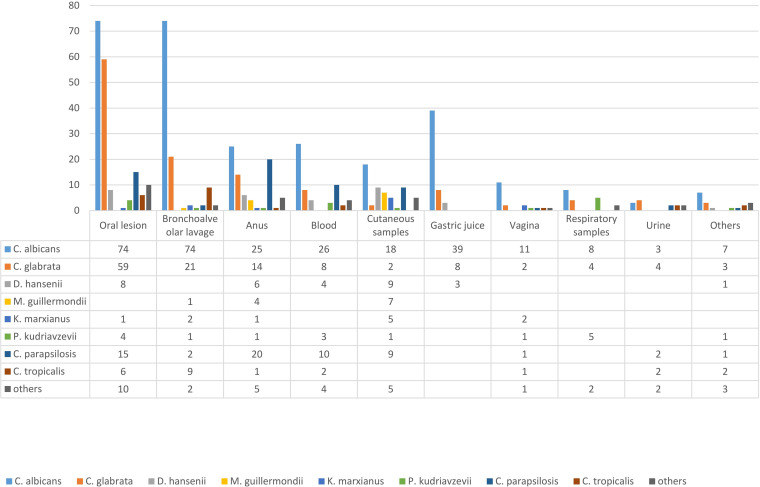
Distribution of specimens from which the *Candida* species has been isolated.

**FIG 2:**
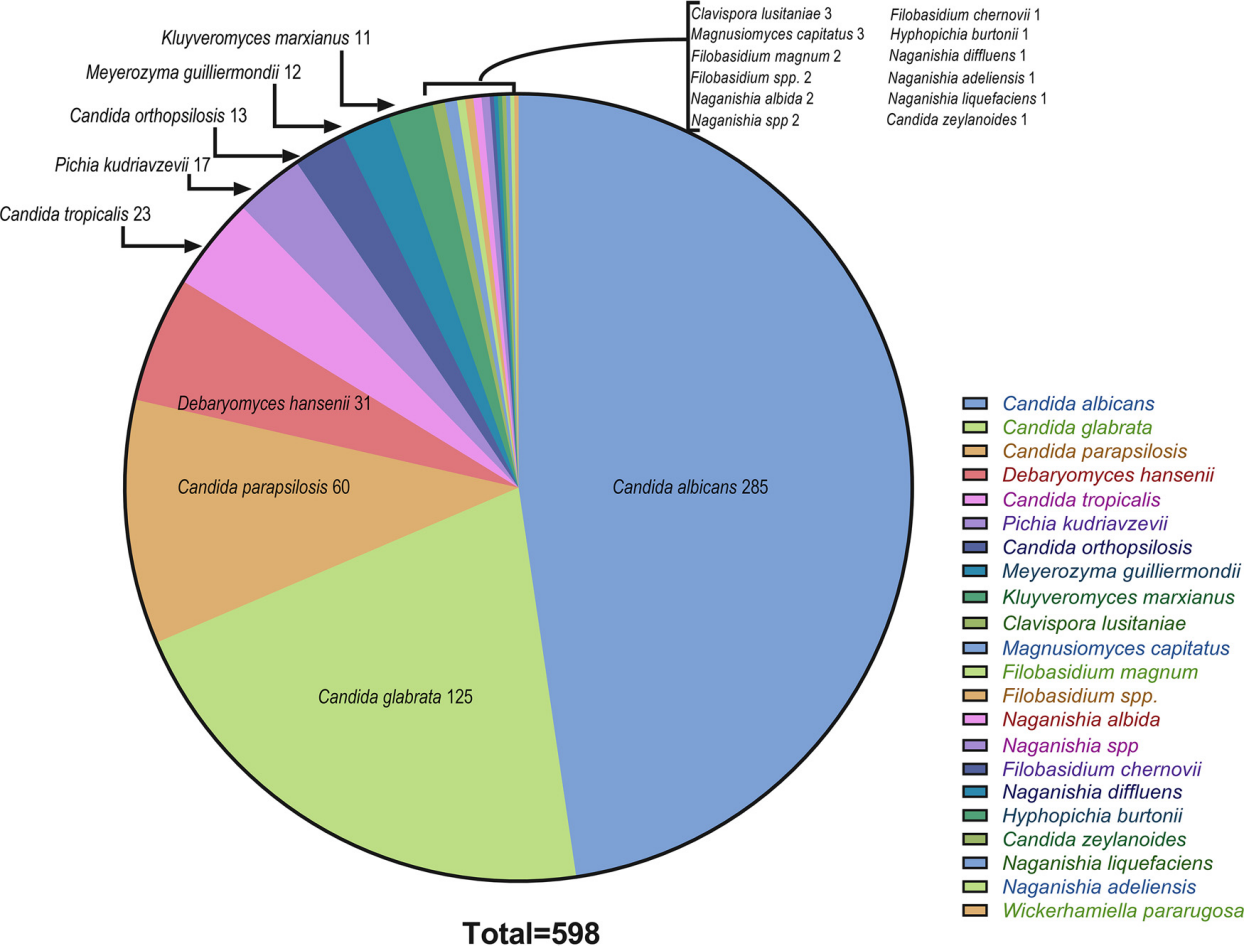
Yeast isolates recovered from 10 university hospitals in Iran.

From 2,385 clinical samples of patients with signs and symptoms of infections, 598 *Candida* isolates were obtained from 10 university hospitals in Iran. Regarding specimen types, 29.6% of the isolates (177/598) were recovered from the oral cavity, 18.6% of isolates (111/598) from bronchoalveolar lavage fluid, 12.9% from the anus (77/598 isolates), 9.5% from blood (57/598 isolates), 9.5% (56/598 isolates) from cutaneous samples (skin and nail), and 8.4% from gastric juice (50/598 isolates). Other specimens included 20 vagina swabs (3.3%); 19 respiratory tract samples, including sinuses and lung tissues (3.2%); 13 urine samples (3.2%); and 18 wounds, abscesses, and cerebrospinal fluids (3%) (Fig. 1). The most commonly isolated *Candida* species was C. albicans (285, 47.7%), followed by C. glabrata (125, 20.9%), C. parapsilosis (60,10%), Debaryomyces hansenii (31.5.2%), C. tropicalis (23, 3.8%), Pichia kudriavzevii (also known as [aka] Candida krusei, 17, 2.8%), C. orthopsilosis (13, 2.2%), Meyerozyma guilliermondii (aka Candida guilliermondii, 12, 2%), Kluyveromyces marxianus (aka Candida kefyr, 11, 1.3%), and Clavispora lusitaniae (aka Candida lusitaniae, 3, 0.5%). Other yeasts identified in this study (4.2%, 25/598) were Torulaspora delbrueckii; Hyphopichia burtonii; Wickerhamiella pararugosa; *Naganishia* species, including N. albida, N. adeliensis, N. diffluens, and N. liquefaciens; Magnusiomyces capitatus; Filobasidium magnum; Filobasidium chernovii; and Candida zeylanoides (Fig. 2).

The antifungal activity data for *Candida* species collected in the current study are presented in Tables 1 and 2. MIC_90_ values in all *Candida* species were as follows: 0.25 μg/mL for caspofungin (CAS) and voriconazole (VOR); 0.5 μg/mL for amphotericin B (AMB) and isavuconazole (ISA); 2 μg/mL for itraconazole (ITR), luliconazole (LUL), and posaconazole (POS); and 16 μg/mL for FLU. In C. albicans species, the MIC_90_ values for voriconazole (VRC), fluconazole (FLU), ITR, POS, LUL, and ISA were 0.125, 4, 0.5, 0.5, 1, and 0.5 μg/mL, respectively (Table 1). The MIC_90_ and epidemiologic cutoff value (ECV) values for AMB and CAS of all *Candida* species were 0.25 and 0.5, and 0.064 and 0.125 μg/mL, respectively. Using interpretative breakpoints defined by the CLSI M60 protocol, 98.5% (281/285), 96.8% (276/285), and 97.9% (279/285) of C. albicans isolates were sensitive to FLU, VRC, and CAS, respectively. Only 1.4%, 1.4%, and 0.7% of the C. albicans species were resistant to FLU, VRC, and CAS, respectively. The non-wild-type (non-WT) phenotype rates regarding AMB, CAS, VRC, FLU, POS, LUL, and ISA for C. albicans were 1.4%, 2.1%, 1.4%, 2.5%, 1.1%, 2.5%, and 2.5%, respectively. In C. albicans species, there was a significant correlation between MIC values of VRC and other antifungal agents (*P* = 0.001). Also, significant correlations were observed between MIC values of FLU, LUL, and ISA.

**TABLE 1:** Comparison of *in vitro* activities of eight antifungal agents (μg/mL) tested against *Candida* species more than 100 isolates by CLSI method^*a*^

*Candida* species	Antifungal agents	Range (mode)	MIC_50_	MIC_90_	MIC_GM_	ECV	WT	Non-WT
Candida albicans (285)	Amphotericin B	0.016–1 (0.125)	0.064	0.25	0.09	0.5	98.6%	1.4%
	Caspofungin	0.016–16 (0.016)	0.016	0.064	0.03	0.25	97.8%	2.1%
	Voriconazole	0.016–8 (0.016)	0.032	0.125	0.36	1	98.6%	1.4%
	Fluconazole	0.064–32 (0.5)	0.5	4	0.76	8	97.5%	2.5%
	Itraconazole	0.016–8 (1)	0.064	0.5	0.08	8	100%	
	Posaconazole	0.016–8 (0.016)	0.064	0.5	0.10	2	98.9%	1.1%
	Luliconazole	0.008–4 (0.5)	0.125	1	0.14	2	97.5%	2.5%
	Isavuconazole	0.008–2 (0.008)	0.016	0.5	0.03	0.5	97.5%	2.5%
Candida glabrata (125)	Amphotericin B	0.016–4 (0.125)	0.25	0.5	0.21	2	98.4%	1.6%
	Caspofungin	0.016–1 (0.064)	0.064	0.125	0.06	0.25	98.4%	1.6%
	Voriconazole	0.016–0.5 (0.125)	0.125	0.25	0.10	0.5	100%	
	Fluconazole	0.125–32 (4)	8	32	5.82	32	100%	
	Itraconazole	0.016–8 (0.25)	0.5	4	0.46	8	100%	
	Posaconazole	0.016–8 (1)	0.25	1	0.58	2	98.6%	1.4%
	Luliconazole	0.008–4 (0.008)	0.032	1	0.05	2	98.4%	1.6%
	Isavuconazole	0.008–0.25 (0.125)	0.064	0.25	0.08	0.5	100%	

a GM, geometric means; ECV, epidemiological cut of value; MIC_50_ and MIC_90_ values (μg/mL), lowest concentration of the antifungal agent at which the growth of 50 and 90% of the isolates were inhibited, respectively.

**TABLE 2:** Comparison of *in vitro* activities of eight antifungal agents (μg/mL) tested against *Candida* species by CLSI method (<100 isolates)

Species	Antifungals	Range (mode)	MIC_50_	MIC_90_	MIC_GM_
Candida parapsilosis (60)	Amphotericin B	0.016–1 (0.25)	0.25	0.5	0.16
	Caspofungin	0.016–2 (0.016)	0.25	1	0.32
	Voriconazole	0.016–0.5 (0.125)	0.032	0.064	0.05
	Fluconazole	0.064–32 (2)	1	4	1
	Itraconazole	0.016–4 (0.125)	0.064	0.25	0.08
	Posaconazole	0.016–2 (0.125)	0.125	0.5	0.11
	Luliconazole	0.008–8 (1)	1	2	0.8
	Isavuconazole	0.008–2 (0.125)	0.032	1	0.06
Debaryomyces hansenii (31)	Amphotericin B	0.016–0.5 (0.25)	0.25	0.5	0.17
	Caspofungin	0.016–4 (4)	0.5	4	0.4
	Voriconazole	0.016–8 (0.016)	0.5	8	0.36
	Fluconazole	0.125–32 (32)	16	32	4.64
	Itraconazole	0.016–8 (8)	1	8	0.68
	Posaconazole	0.016–4 (0.016)	0.5	4	0.3
	luliconazole	0.008–4 (1)	1	4	0.31
	isavuconazole	0.008–2 (0.008)	0.125	0.5	0.08
Candida tropicalis (23)	Amphotericin B	0.064–1 (0.25)	0.25	0.5	0.2
	Caspofungin	0.016–0.125 (0.016)	0.016	0.064	0.02
	Voriconazole	0.016–2 (0.125)	0.064	0.25	0.05
	Fluconazole	0.250–16 (2)	0.25	8	1.53
	Itraconazole	0.016–4 (0.125)	0.064	0.25	0.18
	Posaconazole	0.016–2 (0.125)	0.25	2	0.34
	Luliconazole	0.008–4 (1)	0.5	2	0.45
	Isavuconazole	0.008–2 (0.125)	0.032	1	0.05
Pichia kudriavzevii (17)	Amphotericin B	0.016–1 (0.25)	0.125	1	0.15
	Caspofungin	0.016–2 (0.016)	0.125	2	0.15
	Voriconazole	0.016–0.25 (0.125)	0.125	0.25	0.08
	Fluconazole	0.250–64 (2)	8	32	6.34
	Itraconazole	0.032–2 (0.125)	0.125	2	0.23
	Posaconazole	0.064–1 (0.125)	0.125	1	0.21
	Luliconazole	0.008–2 (1)	0.5	1	0.32
	Isavuconazole	0.008–0.5 (0.125)	0.125	0.25	0.06
Candida orthopsilosis (13)	Amphotericin B	0.016–0.125 (0.25)	0.064	0.064	0.04
	Caspofungin	0.064–0.25 (0.016)	0.125	0.25	0.15
	Voriconazole	0.016–0.064 (0.125)	0.032	0.064	0.03
	Fluconazole	1–2 (2)	2	2	1.6
	Itraconazole	0.125–1 (0.125)	0.125	0.5	0.22
	Posaconazole	0.032–0.5 (0.125)	0.125	0.25	0.15
	Luliconazole	0.064–2 (1)	0.25	2	0.28
	Isavuconazole	0.008–0.032 (0.125)	0.016	0.032	0.1
Meyerozyma guilliermondii (12)	Amphotericin B	0.016–0.5 (0.125)	0.125	0.5	0.09
	Caspofungin	0.016–1 (0.016)	0.25	4	0.17
	Voriconazole	0.016–0.125 (0.016)	0.032	0.125	0.04
	Fluconazole	0.250–16 (0.25)	1	16	1.41
	Itraconazole	0.016–8 (0.5)	0.25	0.5	0.20
	Posaconazole	0.016–8 (0.5)	0.5	8	0.38
	Luliconazole	0.008–1 (0.032)	0.032	0.25	0.05
	Isavuconazole	0.008–0.125 (0.064)	0.064	0.125	0.04
Kluyveromyces marxianus (11)	Amphotericin B	0.016–1 (0.5)	0.25	0.5	0.2
	Caspofungin	0.016–0.125 (0.016)	0.016	0.125	0.03
	Voriconazole	0.016–0.125 (0.016)	0.016	0.125	0.04
	Fluconazole	0.125–0.5 (0.5)	0.5	0.5	0.42
	Itraconazole	0.032–0.25 (0.125)	0.125	0.25	0.12
	Posaconazole	0.016–0.032 (0.016)	0.016	0.032	0.02
	Luliconazole	0.008–0.125 (0.008)	0.008	0.125	0.02
	Isavuconazole	0.008–0.016 (0.008)	0.008	0.016	0.01

The lowest MIC_90_ value of C. glabrata was for CAS (0.125 μg/mL), followed by VRC and LUL (0.250 μg/mL) and AMB (0.5 μg/mL). Approximately 91% (114/125) of C. glabrata isolates were sensitive and 100% were susceptible dose dependent (SDD) to CAS and FLU, respectively. Candida parapsilosis presented MIC_90_ values to AMB, CAS, VRC, FLU, ITR, POS, LUL, and ISA of 0.5 mg/L, 1 mg/L, 0.064 μg/mL, 4 μg/mL, 0.25 μg/mL, 0.5 μg/mL, 2 μg/mL, and 1 μg/mL, respectively. Caspofungin and FLU were effective against C. parapsilosis isolates (100% sensitive), but 53.3% (32/60 isolates) and 11.7% (7/60) of isolates were intermediate and resistant to VOR. The MIC_90_ values for AMB and ISA in D. hansenii (aka Candida famata) were 0.5 μg/mL, while the MIC values for other antifungal agents were high. P. kudriavzevii isolates presented 73.3% (11/15), 86.7% (13/15), and 93.3% (14/15) sensitivity to CAS, VRC, and FLU, respectively. The resistance rate for this organism to CAS and VRC was 13.3% (2/15). The geometrics mean values of FLU, POS, CAS, AMB, VRC, ITR, LUL, and ISA for M. guilliermondii were 1.4, 0.38, 0.17, 0.09, 0.04, 0.2, 0.05, and 0.04, respectively. Totally for all cities, AMB and CAS were the most effective antifungal agents, followed by VRC and ISA.

Although 30/285 of C. albicans (10.5%), 15/31 of D. hansenii (48.4%), 3/12 of M. guilliermondii (25%), 67/125 of C. glabrata (53.6%), 5/15 of P. kudriavzevii (33.3%), 6/60 of C. parapsilosis (10%), and 5/23 of C. tropicalis (21.7%) were multiazole resistant to 2 to 4 azoles, pan-azole resistance was not observed.

## DISCUSSION

Knowledge of regional distributions of causative *Candida* agents and their antifungal susceptibility patterns can help to monitor (emergence of) resistance to antifungal agents of various species and support local and national surveillance programs. *Candida* species occur as commensals in more than half of healthy humans (12) and can cause infections in any part of the human body, like the blood, respiratory system, eyes, and central nervous systems, especially in immunocompromised individuals (13–16). In the present study, C. albicans was the most common yeast (47.7%) isolated from the study population at the participating Iranian university hospitals, followed by C. glabrata, C. parapsilosis, D. hansenii, C. tropicalis, and P. kudriavzevii. In the present study, C. auris have not been isolated from entered patients, but they have been reported in numerous countries on six continents (17).

Candida albicans is the most frequent etiologic agent of candidemia isolated from 39 countries (18). In intensive care unit patients in Mexico, 42.8% of the isolated species were C. albicans, and non-*albicans Candida* species were involved in 57.2% of cases (15). Also, in the latter study, the most prevalent species was C. glabrata, followed by P. kudriavzevii, C. parapsilosis, and C. tropicalis (15). In a study in Brazil, the most isolated species from patients with candidemia were C. parapsilosis (32.6%), followed by C. albicans (27.7%), C. tropicalis (14.6%), and C. glabrata (9.7%) (19). The distribution of species causing candidiasis varies by geographic areas, the patient populations (surgical wards, hematology, ICU, and neonate patients), and hospital care characteristics (20).

Antifungal resistance of *Candida* species may develop after long-term use of antifungal agents for either treatment or prophylaxis (18). The MIC range MIC_50_ and MIC_90_ values of FLU in the present study were 0.016 to 32, 0.5, and 4 μg/mL, respectively. In Brazil, Celestino de Souza et al. (21) studied C. albicans isolated from blood cultures and observed a MIC range of FLU between 0.125 and 1.0 μg/mL, with MIC_50_ and MIC_90_ values of 0.5 μg/mL and 1.0 μg/mL, respectively. In Thailand, the MIC_90_ values of FLU for C. albicans were 1 μg/mL (22). Our results were higher than observed in other studies, likely due to different usage of antifungals and management of patients in Iran.

The increased use of FLU for either treatment or prophylaxis of immunocompromised patients may be associated with a rise in infections caused by C. glabrata (18). In the present study, non-WT phenotype isolates of C. glabrata for AMB, CAS, POS, and LUL were observed, and all isolates were found to be resistant to FLU. Rodrigues and coworkers (19) reported non-WT species in Brazil of 28.6% (4/14) isolates of C. glabrata for FLU and 28.6% (4/14) of isolates for VRC. High numbers of FLU-resistant C. glabrata have been reported in the United States, Australia, Denmark, and Belgium (23, 24). Multidrug resistance has been reported in echinocandins accompanied by azole resistance among C. glabrata species (25, 26). About 28.6% of C. glabrata isolates from Brazil were non-WT to VRC, and all were resistant to FLU (19). Candida tropicalis is one of the candidemia causative agents with high mortality rates (27). In a study by Chong and coworkers (28) on fatal candidemia in hematological malignancies patients caused by C. tropicalis, a significant increase in the number of azoles and AMB-resistant C. tropicalis was reported. In a study by Arastehfar and coworkers (27), resistance to VRC and FLU was observed in seven (10.93%, 7/64) and four (6.25%, 4/64) C. tropicalis isolates, respectively. Cross-resistance to VRC, ITR, and POS was observed in 28.57% of FLU-resistant C. tropicalis isolates in Thailand (22). In Boonsilp's study in Thailand, all C. tropicalis isolates were susceptible to CAS (22). In the present study, all C. tropicalis were sensitive to CAS, VRC, and FLU, according to the CLSI M60 protocol. The difference in susceptibility to different antifungals is likely due to the managing the use of antifungal drugs in each region.

Candida parapsilosis is mostly isolated from premature newborns with low birth weight (18). In the present study, the MIC_90_ value for FLU was 4 μg/mL and most isolates were susceptible doses depending according to CLSI M60. Approximately 6.4% of clinical C. parapsilosis isolates from 22 hospitals in São Paulo State presented poor susceptibility to FLU (19). The MIC range of FLU in C. parapsilosis complex isolates from the United States was 0.25 to 4.0 μg/mL, and MIC_50_ and MIC_90_ values were 1 and 2 μg/mL, respectively (29). In a study in Thailand, the MIC_90_ values for AMB, POS, FLU, ITR, and VRC of C. parapsilosis were 0.5, 0.12, 2, 0.25, and 0.12 μg/mL, respectively (22). The frequencies of M. guilliermondii in Brazil and Thailand were 1.4% (2/144) (24) and (1.85%) (22), respectively, and in agreement with the present study (2%). In the largest candidemia study performed in South and Central America, the frequency of M. guilliermondii was 20.7% in Honduras (30). In Boonsilp et al. (22), M. guilliermondii was susceptible to CAS, but it displayed reduced susceptibility to POS. Evaluation of the susceptibility patterns of *Candida* species to antifungal agents in different geographical regions can optimize the treatment of candidiasis. Our results are different from those previously published and likely due to different management of patient treatment and prophylaxis in each region.

The current study has some limitations as our study included isolates from only 10 university hospitals in Iran, which may not be representative of the entire country. However, our findings present valuable data about the prevalence of *Candida* etiologic agents and susceptibility data of the isolates. Resistant species and isolates might be restricted to geographic regions; therefore, extensive surveillance studies should be conducted to gain knowledge about the local epidemiology of *Candida* species and their antifungal resistance rates and susceptibility patterns to antifungal agents, including new ones.

According to the present study results, C. albicans and C. glabrata were the most frequently isolated species from clinical samples in Iran. Increasing rates of non-*albicans Candida* isolates from the Iranian population, should be looked at as alarming due to various levels of intrinsic MIC values or resistance to various antifungal drugs. Caspofungin and VRC are recommended over FLU for the treatment of *Candida* infections in the study region. However, AMB and ISA are also active against the most common *Candida* species isolated from patients. Pan azole-resistant *Candida* species were not observed in the present study.

## MATERIALS AND METHODS

The study was approved by the ethics committee of the National Institute for Medical Research Development (IR.NIMAD.REC.1398.319).

### Sample collection.

All *Candida* isolates obtained from patients with signs and symptoms of fungal infections were included in the present study. During the study period from Dec 2019 to Dec 2021, *Candida* isolates were collected from clinical samples of patients admitted to 10 tertiary care Medical University Hospitals in Iran (i.e., Shiraz, Ahvaz, Isfahan, Kerman, Mashhad, Khorram Abad, Sanandaj, Urmia, Yasuj, and Zahedan). Clinical samples (i.e., sinuses, lung tissue, blood, oral lesions, pleural tap, bronchoalveolar lavage, sputum, vagina, and cutaneous samples) were cultured on Sabouraud dextrose agar plates (Merck, Germany) and incubated at 22 to 25°C for 3 to 5 days.

### Molecular identification.

DNA extraction from *Candida* isolates was performed according to Lõoke et al. (31). One loop of the isolated yeasts was suspended in 100 μL lithium acetate–SDS solution (200 mM LiOAc 1% SDS) and incubated for 15 min at 70°C. DNA was precipitated by adding 300 μL ethanol 96%. For molecular identification of common *Candida* species, we prepared the PCR mixture containing 10× reaction buffer (5 μL), MgCl_2_ (1.5 mM), dNTPs (0.4 mM), DNA *Taq* polymerase (2.5 U), and 30 pmol of each ITS1 (5′-TCCGTA GGTGAACCTGCG G-3′) and ITS4 (5′-TCCTCCGCT TATTGATATGC-3′) primers and extracted DNA (10 μL) in a final volume of 50 μL. The PCR conditions were according to Mirhendi et al. (32). The PCR products were digested with the MspI restriction enzyme. To evaluate the lengths of amplified products and restriction fragments, a 50 bp DNA ladder (GeneRuler, Fermentas, Lithuania) was used, and the gels were analyzed under UV light using a gel documentation system (BioCell Azma, Cell Aria gel imaging system, Iran). The amplified products were visualized by electrophoresis after running in 1.5% agarose gels for an hour. The digested fragments of the restriction fragment length polymorphism reaction were run on a 2% agarose gel. Also, the PCR products of isolates were identified by sequencing. The obtained data were compared to the NCBI nucleotide database (BLAST; https://blast.ncbi.nlm.nih.gov/Blast.cgi).

### Antifungal susceptibility testing.

The antifungal susceptibility tests of each isolate to AMB, CAS, VRC, fluconazole (FLU), POS, ITR, LUL, and ISA were performed according to the microdilution CLSI M27, M59, and M60 methods (33–35). The powders of antifungal agents were obtained from the following manufacturers: VRC, FLU, ITR, and CAS from Sigma, USA and POS and AMB from Sigma, Germany. The concentration range of VRC, FLU, POS, ITR, CAS, and AMB was 0.03 to 16 μg/mL and for LUL and ISA, this was 0.008 to 8 μg/mL. Quality control was ensured using C. parapsilosis ATCC 22019. The MICs of AMB were reported as the lowest drug concentration that lacked any visual growth (100%). For POS, FLU, VRC, CAS, and ITR, the lowest concentration inhibiting the growth by 50% compared to positive controls was taken as MIC.

### Statistical analysis.

Data were collected using SPSS version 16. The MIC ranges (MIC_50_ and MIC_90_) and geometrics means (MIC_GM_) for each *Candida* species were calculated. Epidemiologic cutoff values and WT and non-WT species were calculated for those *Candida* species with more than 100 isolates by the eyeball method (36). Correlations between the MIC values of the antifungal agents and *Candida* species were evaluated by the Pearson correlation test using a significance level of 0.05.

### Data availability.

All sequences generated in the current study were deposited in GenBank (https://www.ncbi.nlm.nih.gov/genbank/) under the following accession numbers. C. parapsilosis (OM756731-OM756733, OM801503-OM801510, OM801513, ON159295, OK298402-OK298409, OK303405, OK303407-OK303416, OK305953-OK305957, OK310778, OK310779, OK310784-OK310787, OK317692, OK317693, OK618665); C. orthopsilosis (OK305956, OK298481-OK298488, OK310780-OK310783), C. albicans (OK618520, OK618521, OK305936, OK310777, OK305934); C. glabrata (OK317687, OK317691), M. guilliermondii (OK481121); C. tropicalis (OK303417, OK305935, OK305947); Clavispora lusitaniae (OK303418, OK305946); Kluyveromyces marxianus (OK303406, OK305948, OM756710); Torulaspora delbrueckii (OM756728); Pichia kudriavzevii (OK305952, OK317694, OK317695); Hyphopichia burtonii (OK303419); Wickerhamiella pararugosa (OK303420), *Naganishia* species, including N. albida (OK305958, OK305951 and OK317688), N. adeliensis (OK305933), N. diffluens (OK305959), and N. liquefaciens (OK305932); Magnusiomyces capitatus (OK310776); Filobasidium magnum (OK317686, OK317685); and Filobasidium chernovii (OM756729).
